# Age- and sex-dependency of the association between systemic antioxidant potential and glaucomatous damage

**DOI:** 10.1038/s41598-017-08624-4

**Published:** 2017-08-14

**Authors:** Yoshimi Asano, Noriko Himori, Hiroshi Kunikata, Mai Yamazaki, Yukihiro Shiga, Kazuko Omodaka, Hidetoshi Takahashi, Toru Nakazawa

**Affiliations:** 1 Department of Ophthalmology, Tohoku University Graduate School of Medicine, Sendai, Japan; 2 Department of Retinal Disease Control, Tohoku University Graduate School of Medicine, Sendai, Japan; 3 Department of Ophthalmic Imaging and Information Analytics, Tohoku University Graduate School of Medicine, Sendai, Japan; 4 Division of Ophthalmology Tohoku Medical and Pharmaceutical University Department of Medicine, Sendai, Japan; 5 Department of Advanced Ophthalmic Medicine, Tohoku University Graduate School of Medicine, Sendai, Japan

## Abstract

Systemic oxidative stress is thought to be an important factor in the pathogenesis of glaucoma. In particular, low systemic antioxidative capacity, which normally counters oxidative stress, may contribute to glaucoma. Thus, we investigated the association between biological antioxidant potential (BAP), a biomarker of systemic antioxidative capacity, and glaucoma severity in patients with open-angle glaucoma (OAG). This study included 480 eyes of 240 patients with OAG and 66 healthy control eyes. We measured the BAP serum level with a free radical analyzer and compared it with a weighted estimate of the number of retinal ganglion cells (wrgc), derived from circumpapillary retinal nerve fiber layer thickness and visual field mean deviation. We found that wrgc was uncorrelated with BAP in the overall, male, and female OAG patients, but was correlated in young (aged ≤ 65 years) male OAG patients (better eye: *r* = 0.33, *P* = 0.02; worse eye: *r* = 0.27, *P* = 0.047). Furthermore, a mixed-effects regression analysis revealed that BAP was an independent contributing factor to wrgc in young male OAG patients (*P* = 0.02). Thus, systemic antioxidant capacity was associated with glaucomatous damage in relatively young male patients, suggesting that anti-oxidant therapy might be more effective in these patients.

## Introduction

Glaucoma is characterized by the progressive loss of retinal ganglion cells (RGCs)^1^. The most important risk factor for glaucoma is high intraocular pressure (IOP), although the disease may progress despite IOP-lowering treatments^2^. This has prompted ongoing research into the molecular mechanisms of glaucomatous retinal neurodegeneration, with the final aim of identifying biomarkers of glaucoma progression and developing neuroprotective treatment strategies. Candidate molecular mechanisms of neurodegeneration include oxidative stress^3^, toxicity caused by excitatory amino acids, such as glutamate^4^, toxicity caused by nitric oxide^5^, aging^6^, and decreased ocular blood flow^7^. Among these factors, oxidative stress may be the most promising source of new biomarkers and treatments^8–11^.

Oxidative stress is caused by an imbalance between the production of reactive oxygen species (ROS) and their elimination by antioxidants. Even if oxidative stress levels are normal, an insufficient antioxidant level can result in oxidative stress-induced damage to a variety of macromolecules, such as proteins, DNA, and lipids. However, despite a great deal of recent research into the role of oxidative stress in glaucoma, few studies have evaluated the association between glaucoma severity and systemic antioxidant status. Previously, we studied the relationship between glaucoma and skin autofluorescence (SAF), a biomarker of oxidative stress that reflects the skin level of accumulated advanced glycation end products (AGEs). SAF is known to be correlated with the presence of chronic diseases, such as diabetes mellitus^12^. Our investigation showed that SAF was most strongly associated with glaucomatous visual field deterioration in relatively young patients^9^, suggesting that the role of oxidative stress in glaucoma may vary with the age of the patient, and that the potential benefit of anti-oxidant treatments might therefore also vary. Another previous study of oxidative stress biomarkers in glaucoma patients, by Tanito *et al*., found that the presence of open-angle glaucoma (OAG) in patients was related to low levels of biological antioxidant potential (BAP), an indicator of the serum antioxidant capacity level^13–15^. Taking these previous findings into consideration, we therefore set out to investigate the effect of age on the relationship between the level of BAP and glaucoma severity.

In this study, we chose a weighted estimate of surviving RGCs (i.e., weighted RGC count: wrgc) to estimate glaucoma severity. Another parameter of glaucoma severity, circumpapillary retinal nerve fiber layer thickness (cpRNFLT), has been shown to reflect significant early structural changes that occur in many glaucoma patients. Moreover, these changes precede detectable changes in Humphrey Field Analyzer (HFA)-measured mean deviation (MD), a functional measurement. However, although structural measurement may thus be a useful tool in early glaucoma, their usefulness in moderate and advanced glaucoma has been questioned^16–18^. In these later stages of glaucoma, MD may be a better method to quantify severity and monitor progression. This situation prompted the recent development of wrgc, which is a combined staging system that can assess glaucoma severity based on the number of surviving RGCs. This estimated number is based on a weighted calculation that includes both cpRNFLT and MD. Wrgc has been found to improve the reliability and accuracy of estimates of the amount of neural losses, and Yamazaki *et al*. reported that wrgc is potentially a valuable indicator of glaucoma severity over a wide range of disease stages^19^.

Thus, the current study compared BAP, a biomarker of systemic antioxidant capacity that can easily be measured clinically, and wrgc, a marker of glaucoma severity that can be derived from already available ophthalmological findings in patients with OAG. Furthermore, we attempted to determine whether systemic parameters, such as age and sex, influenced BAP, and evaluated the relationship between BAP and wrgc in eyes with OAG, in order to uncover new details on the role of systemic antioxidant capacity in the development and progression of glaucoma, and test our hypothesis that oxidative stress has a particularly important role in younger patients.

## Results

### Clinical characteristics of the control subjects and OAG patients

Two hundred and forty Japanese patients with glaucoma (106 male and 134 female) and 66 normal subjects (24 male and 42 female) were recruited into this study. The clinical characteristics of the control and OAG patients are shown in Table 1. Overall, there were significant differences between the control and OAG patients in visual acuity, axial length of both the better and worse eyes, IOP in the worse eye (*P* < 0.01, *P* < 0.01, *P* = 0.01, *P* = 0.02), and the incidence of diabetes (*P* = 0.01). Among the male subjects, BAP was lower in the OAG patients than in the control subjects (*P* < 0.01), while there were no significant differences in the incidence of hyperlipidemia, diabetes, or a current smoking habit. Among the female subjects, although there was no significant difference in BAP between the OAG and control subjects, there were significant differences in visual acuity, axial length, and IOP in both the better and worse eyes (better-eye; *P* < 0.01, *P* = 0.04, *P* = 0.04, worse-eye; *P* = 0.02, *P* = 0.04, *P* = 0.04).

**Table 1:** Characteristics of OAG and normal control.

	All					Male					Female				
	control	OAG		*P value*		control	OAG		P value		control	OAG		*P* value	
				control vs OAG					control vs OAG					control vs OAG	
		better eye	worse eye	better eye	worse eye		better eye	worse eye	better eye	worse eye		better eye	worse eye	better eye	worse eye
Number of patients	66	240	240	−	−	24	106	106	−	−	42	134	134	−	−
NTG: POAG	−	168:72	−	−	−	−	70:36	−	−	−	−	98:36	−	−	−
Age (yrs)	58.9 ± 16.7	64.5 ± 11.7	−	0.06	−	58.2 ± 16.2	64.1 ± 12.0	−	0.14	−	59.3 ± 17.1	64.9 ± 11.6	−	0.19	−
VA (logMAR)	0.04 ± 0.2	−0.01 ± 0.2	0.08 ± 0.4	<0.01	0.14	−0.02 ± 0.1	−0.01 ± 0.2	0.1 ± 0.3	0.13	0.54	0.08 ± 0.2	−0.02 ± 0.2	0.1 ± 0.4	<0.01	0.02
AL (mm)	23.9 ± 1.3	24.4 ± 1.3	24.4 ± 1.3	<0.01	0.01	24.4 ± 1.3	24.7 ± 1.2	24.7 ± 1.2	0.36	0.3	23.7 ± 1.2	24.2 ± 1.3	24.2 ± 1.3	0.04	0.04
IOP (mmHg)	14.5 ± 2.8	13.8 ± 3.5	13.9 ± 4.4	0.06	0.02	14.3 ± 3.4	13.9 ± 3.8	14.4 ± 5.2	0.53	0.33	14.6 ± 2.4	13.7 ± 3.3	13.6 ± 3.6	0.04	0.04
MD (dB)	−	−9.2 ± 7.4	−15.8 ± 8.2	−	−	−	−9.3 ± 7.5	−17.0 ± 8.2	−	−	−	−9.2 ± 7.4	−14.8 ± 8.0	−	−
wrgc (x1000cells)	−	533.1 ± 258.1	372.1 ± 247.3	−	−	−	527.4 ± 269.4	340.9 ± 260.4	−	−	−	537.7 ± 249.7	386.8 ± 234.6	−	−
dROM (U.CARR)	350.5 ± 54.8	351.3 ± 62.1	−	0.89	−	324.4 ± 44.92	340.4 ± 62.4	−	0.29	−	365.4 ± 54.8	359.9 ± 60.7	−	0.46	−
BAP (mmol/L)	2103.7 ± 218.5	2041.0 ± 244.0	−	0.09	−	2197.4 ± 236.6	2020.3 ± 261.6	−	<0.01	−	2050.2 ± 190.2	2059.1 ± 229.2	−	0.74	−
BMI (kg/m^2^)	23.1 ± 3.3	23.0 ± 3.3	−	0.79	−	22.7 ± 2.4	23.8 ± 3.2	−	0.16	−	23.3 ± 3.8	22.3 ± 3.3	−	0.25	−
HL (%)	14 (21.2)	43 (18.1)	−	0.59*	−	4 (16.7)	18 (17.0)	−	1.00*	−	10 (23.8)	25 (18.9)	−	0.51*	−
Diabetes (%)	2 (3.0)	34 (14.3)	−	0.01*	−	1 (4.2)	22 (20.8)	−	0.07*	−	1 (2.4)	12 (9.1)	−	0.19*	−
Current smoker (%)	5 (7.6)	25 (10.5)	−	0.64*	−	4 (16.7)	21 (19.8)	−	1.00*	−	1 (2.4)	4 (3.0)	−	1.00*	−

OAG = open-angle glaucoma, NTG = normal tension glaucoma, POAG = primary open angle glaucoma, VA = visual acuity, logMAR = logarithm of minimum angle of resolution, IOP = intraocular pressure, MD = mean deviation, AL = axial length, BMI = body mass index, dROM = diacron reactive oxygen metabolites, BAP = biological antioxidant potential, U. Carr = carrelli units, HL = hyperlipidemia, wrgc = weighted scale combining the estimated retinal ganglion cell count.

Unmarked P values: Mann-Whitney U test, *Fisher exact test.

### Clinical characteristics of the ≤65-YO OAG patients

Table 2 shows the clinical characteristics of the ≤65-YO OAG patients. A total of 124 of the 240 OAG patients fell into this group. There were no significant differences in dROM or BAP between the ≤65-YO male and female OAG patients. However, there were significant differences in the rate of diabetes and a current smoking habit between the ≤65-YO male and female OAG patients (all *P* < 0.01).

**Table 2:** Comparison between male and female in OAG patients ≤65 yrs.

	≤65 yrs OAG better eye			*P*-value	≤65 yrs OAG worse eye		
	All	Male	Female	Male vs Female	All	Male	Female
Number of patients	124	54	70	−	124	54	70
Age (yrs)	55.6 ± 8.4	55.1 ± 9.0	56.0 ± 7.9	0.86	−	−	−
Sex (Male:Female)	54:70	54:0	0:70	−	−	−	−
NTG:POAG	88:36	38:16	50:20	0.84*	−	−	−
dROM (U.CARR)	343.8 ± 55.9	339.8 ± 59.7	347.0 ± 52.9	0.33	−	−	−
BAP (mmol/L)	2042.5 ± 240.7	2016.5 ± 267.3	2063.0 ± 217.4	0.45	−	−	−
AL (mm)	24.9 ± 1.1	25.0 ± 1.1	24.9 ± 1.1	0.38	24.9 ± 1.1	25.0 ± 1.1	24.8 ± 1.1
IOP (mmHg)	13.4 ± 3.4	14.1 ± 3.6	13.6 ± 3.5	0.32	14.3 ± 4.2	15.0 ± 4.5	13.8 ± 3.8
VA (logMAR)	−0.1 ± 0.2	−0.1 ± 0.2	−0.04 ± 0.2	0.15	0.03 ± 0.3	0.05 ± 0.35	0.01 ± 0.3
CpRNFLT (mm)	83.3 ± 13.8	83.3 ± 14.9	83.3 ± 12.9	0.77	78.0 ± 13.5	76.3 ± 13.9	79.4 ± 13.2
MD (dB)	−8.4 ± 7.3	−8.3 ± 7.6	−8.57 ± 7.11	0.64	−15.0 ± 8.1	−15.8 ± 8.5	−14.5 ± 7.8
wrgc × 1000 cells	599.1 ± 268.3	614.5 ± 297.8	587.1 ± 244.11	0.76	426.6 ± 266.0	415.0 ± 301.6	435.7 ± 236.4
HL (%)	19 (15.3)	7 (12.7)	12 (17.4)	0.62*	−	−	−
Diabetes (%)	15 (12.1)	12 (21.8)	3 (4.3)	<0.01*	−	−	−
Current smoker (%)	16 (12.8)	13 (23.6)	3 (4.3)	<0.01*	−	−	−

OAG = open−angle glaucoma, NTG = normal tension glaucoma, POAG = primary open angle glaucoma, VA = visual acuity, logMAR = logarithm of minimum angle of resolution, IOP = intraocular pressure, MD = mean deviation, AL = axial length, BMI = body mass index, dROM = diacron reactive oxygen metabolites, BAP = biological antioxidant potential, U. Carr = carrelli units, HL = hyperlipidemia, wrgc = weighted scale combining the estimated retinal ganglion cell count, cpRNFLT = circumpapillary retinal nerve fiber layer thickness.

Unmarked *P* values: Mann-Whitney U test, *Fisher exact test.

### Association between wrgc and BAP

**Figure 1:**
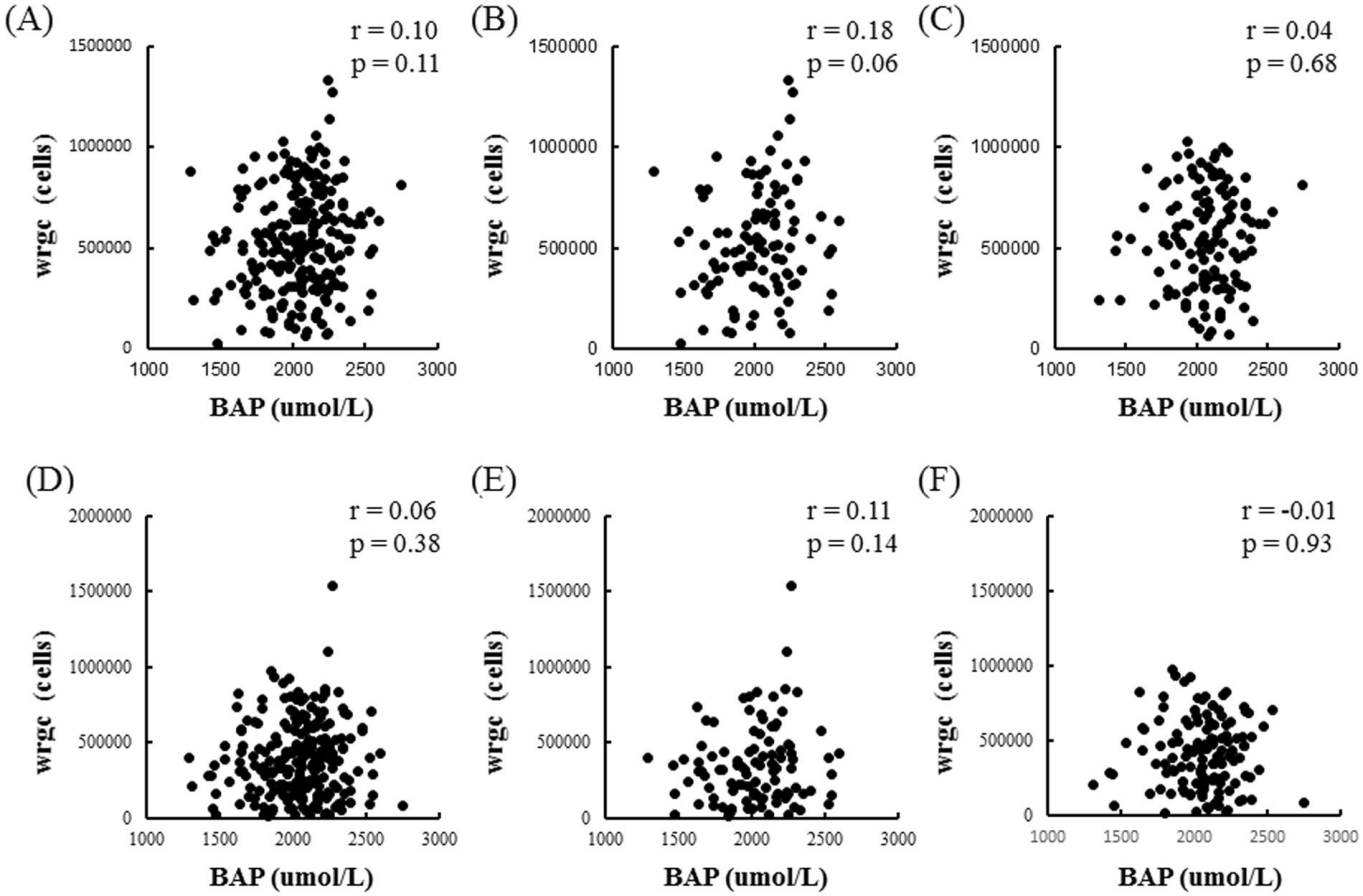
The relationship between BAP and wrgc in OAG patients. (**A**) Better-eye wrgc was not correlated with BAP in OAG patients (r = 0.10, *P* = 0.11). (**B**) Better-eye wrgc was not correlated with BAP in male OAG patients (r = 0.18, *P* = 0.06). (**C**) Better-eye wrgc was not correlated with BAP in female OAG patients (r = 0.04, *P* = 0.68). (**D**) Worse-eye wrgc was not correlated with BAP in OAG patients (r = 0.06, *P* = 0.38). (**E**) Worse-eye wrgc was not correlated with BAP in male OAG patients (r = 0.11, *P* = 0.14). (**F**) Worse-eye wrgc was not correlated with BAP in female OAG patients (r = −0.01, *P* = 0.93).

**Figure 2:**
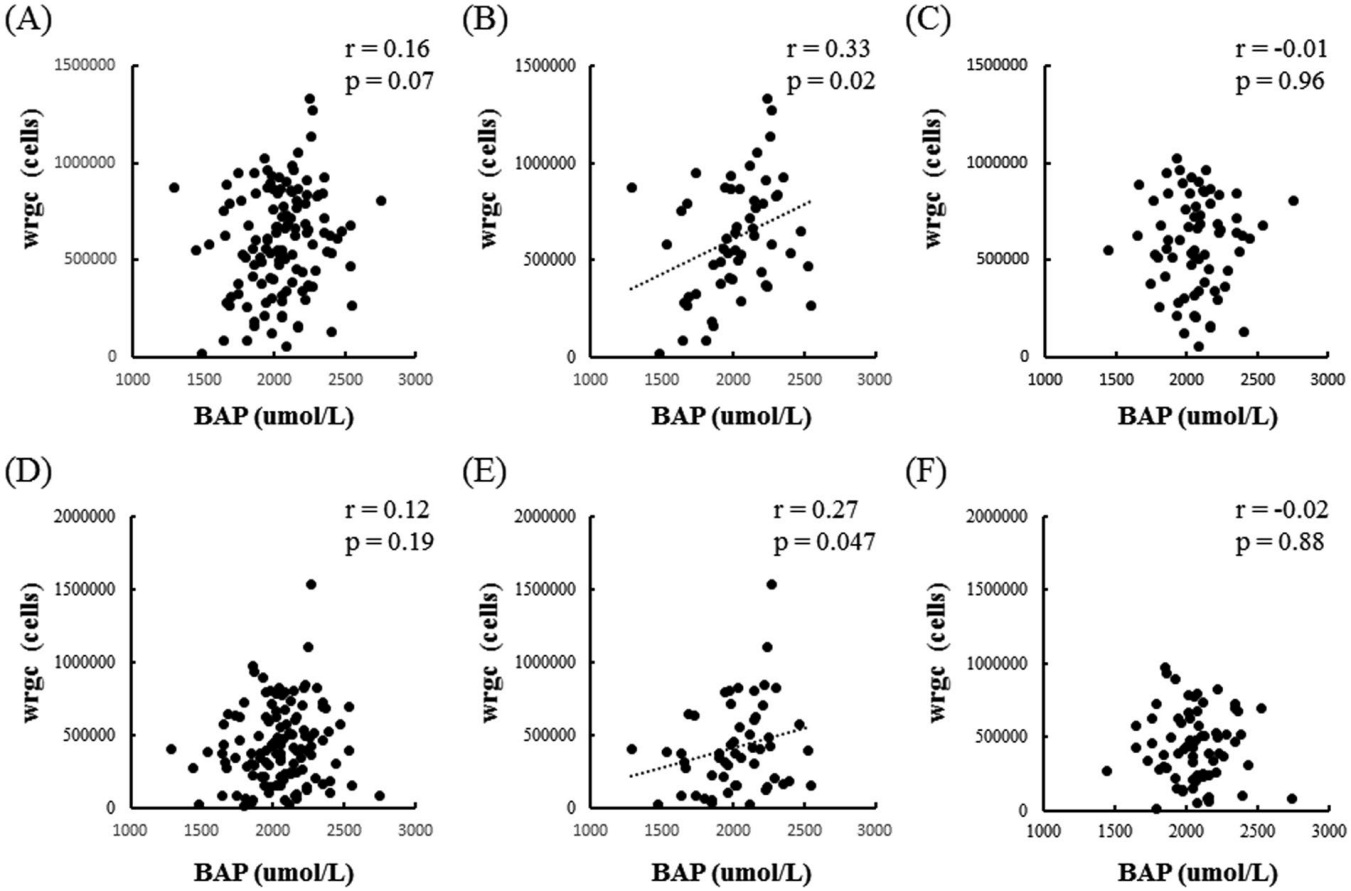
The relationship between BAP and wrgc in OAG patients ≤65 YO. (**A**) Better-eye wrgc was not correlated with BAP in the overall group of OAG patients ≤65 YO (r = 0.16, *P* = 0.07). (**B**) Better-eye wrgc was correlated with BAP in male OAG patients ≤65 YO (r = 0.33, *P* = 0.02). (**C**) Better-eye wrgc was not correlated in female OAG patients ≤65 YO (r = −0.01, *P* = 0.96). (**D**) Worse-eye wrgc was not correlated with BAP in the overall group of OAG patients ≤65 YO (r = 0.12, *P* = 0.19). (**E**) Worse-eye wrgc was correlated with BAP in male OAG patients ≤65 YO (r = 0.27, *P* = 0.047). (**F**) Worse-eye wrgc was not correlated in female OAG patients ≤65 YO (r = −0.02, *P* = 0.88).

Better- and worse-eye wrgc was uncorrelated with BAP in the OAG patients overall and in male- and female-only subgroups (Fig. 1A–F). BAP was also uncorrelated with better-eye wrgc in a group combing the ≤65-YO male and female OAG patients (Fig. 2A). Interestingly, however, BAP was correlated with better-eye wrgc in a group containing only ≤65-YO male OAG subjects (r = 0.33, *P* = 0.02, Fig. 2B) and uncorrelated with better-eye wrgc in the ≤65-YO female OAG subjects (Fig. 2C). Worse-eye wrgc was uncorrelated with BAP in the ≤65-YO OAG patients (Fig. 2D). However, worse-eye wrgc was correlated with BAP in the ≤65-YO male OAG subjects (r = 0.27, *P* = 0.047, Fig. 2E) and was uncorrelated in the ≤65-YO female OAG subjects (Fig. 2F).

### Multiple regression analysis

The results of a multiple regression analysis are shown in Table 3. CpRNFLT, age, and VA were independent contributing factors to wrgc in the all OAG patients (all factors *P* < 0.01, Table 3A). CpRNFLT, BAP, IOP, and age were independent contributing factors to wrgc in the ≤65-YO male OAG patients (*P* < 0.01, *P* = 0.02, *P* = 0.04, and *P* < 0.01, respectively, Table 3B).

**Table 3:** Coefficient estimates of linear mixed model for mean deviation in open-angle glaucoma patients.

Variable		β	*P* value
Dependent	Independent		
**(A)**			
wrgc	Age (yrs)	−4350.4	<0.01
	VA (logMAR)	−143675.8	<0.01
	IOP (mmHg)	1884.7	0.32
	BAP (mmol/L)	55	0.08
	CpRNFLT (mm)	11876.9	<0.01
**(B)**			
wrgc	Age (yrs)	−6943.5	<0.01
	VA (logMAR)	−105369.9	0.053
	IOP (mmHg)	7894.9	0.04
	BAP (mmol/L)	144.9	0.02
	CpRNFLT (mm)	15176.1	<0.01

VA = visual acuity, logMAR = logarithm of minimum angle of resolution, IOP = intraocular pressure, BAP = biological antioxidant potential, U. Carr = carrelli units, wrgc = weighted scale combining the estimated retinal ganglion cell count, cpRNFLT = circumpapillary retinal nerve fiber layer thickness.

## Discussion

This study examined a large group of patients with glaucoma, and set out to determine whether clinical findings in these patients were associated with two biomarkers related to systemic oxidative stress: BAP, a biomarker of systemic antioxidant levels; and dROM, an oxidative stress biomarker. We evaluated the male and female subjects separately, because of well-known sex-based differences in anti-oxidant and oxidative stress levels. Furthermore, based on a previous study in which we found that measurements of SAF, a skin-based indicator of accumulated oxidative stress, were more highly correlated with glaucoma severity in relatively younger patients^9^, we also performed a separate analysis of the relatively young patients, i.e., those aged less than 65 years. This study design led us to the novel finding that relatively young male OAG patients showed a correlation between BAP and wrgc (in a single regression analysis) and that BAP was also an independent contributing factor to wrgc (in a multiple regression analysis) in this group. Thus, sex and age may have important influences on the role of oxidative stress in the pathogenesis of glaucoma, and relatively young, male OAG patients with low systemic antioxidant levels may have a particularly high susceptibility to oxidative stress, resulting in glaucoma progression.

One of the two key findings of this study was that BAP and wrgc were related in younger male patients, but not in younger female patients. A related finding was that the male control subjects and male glaucoma patients also had significant differences in BAP levels. Although the younger male and female patients had a significantly different rate of diabetes (Table 2), BAP levels did not differ in these patients, suggesting that differences in the rate of diabetes should not have affected the analysis. Many previous reports have suggested that it is necessary to analyze biomarkers of oxidative stress separately in male and female subjects. One study found that dROM and BAP were higher in female than male subjects^20^. We previously found that 8-hydroxy-2′-deoxyguanosine levels were correlated with ocular blood flow^8^. We also found that ocular blood flow, especially in the vessel area of the optic nerve head, was higher in female than male subjects^21^. Finally, a significantly higher proportion of patients with advanced glaucoma are men^22^. Thus, we performed separate analyses of the male and female subjects. Sex-based differences in oxidative stress levels and glaucoma might be related to female hormones, such as estradiol and estrogen, which may have a preventative effect against neurodegenerative diseases, most likely via activation of the antioxidant defense system^23^. Studies have also demonstrated that estrogen has a neuroprotective effect against RGC death^24, 25^. Since female subjects, especially younger female subjects, are less susceptible to oxidative stress, it may not be surprising that they showed a lower correlation between BAP and wrgc. Thus, our findings, as well as those previously reported by others, suggest that a possible underlying mechanism for our finding that male subjects were more susceptible to oxidative stress.

The second key finding of this study was that younger age strengthened the correlation of BAP with wrgc. This reinforces our previous finding that SAF was negatively correlated with MD in a younger group of patients, but not in an older group^9^. Normally, younger people have lower levels of systemic oxidative stress, as shown by Higashi *et al*.^26^. Indeed, while most of the younger patients in this study had normal BAP, a subset had low BAP with correspondingly low wrgc. This suggests that the normal role of antioxidants in protecting the RGCs may have become altered in these patients, possible due to such previously reported factors as a low serum level of anti-oxidant proteins^27^. Thus, in addition to sex, age also could alter the relationship between BAP and wrgc. Taken together, our findings therefore suggest that younger patients with glaucoma have impaired systemic antioxidative stress capacity, and that protection against oxidative stress is most important in these patients.

In the current study, in contrast to the results for BAP, there were no significant differences in dROM between the controls and any of the OAG subgroups, nor were there any associations with wrgc in a multiple regression analysis. Thus, though it is still difficult to evaluate systemic imbalances between ROS and antioxidant levels, we consider that BAP has great potential for future use as a predictive biomarker of oxidative stress-induced glaucomatous damage, particularly under circumstances when the production of ROS is normal.

A great deal of recent research in human subjects indicates that systemic oxidative stress is involved with glaucomatous optic neuropathy (GON), although the precise mechanism remains unclear, while animal-based research has shown that anti-oxidant treatments have a neuroprotective effect in the RGCs. A recent study of human glaucoma subjects showed that low glutathione levels were associated with higher susceptibility to glaucoma^28^, suggesting that the mechanism of oxidative stress involvement in glaucoma may involve a low level of serum anti-oxidant proteins. This is corroborated by findings that glaucoma risk is associated with a low intake of green vegetables ^29^, and that extract of *Ginkgo biloba*, a nitric oxide scavenger, had a protective effect in some patients^30^. Finally, a prospective population-based study revealed that dietary intake of anti-oxidant nutrients protected against glaucoma^29, 31^. Among animal-based studies, recent work has shown that mice with ocular hypertension that received treatment with Tempol, a multifunctional antioxidant, underwent decreases in the activation of NF-κβ and in the production of cytokines in the retina and optic nerve, when compared to vehicle-treated controls^32^. Additionally, treatment with coenzyme Q10 has been shown to inhibit oxidative stress-induced mitochondrial alterations in DBA/2J mice with hereditary glaucoma^33^. In a rat model of glaucoma, overexpression of thioredoxins protected the RGCs^34^. Thus, animal-based studies indicate that antioxidant treatment can decrease oxidative stress and improve neuron survival in glaucoma. Our present study builds on these previous reports with the finding that systemic antioxidant levels are closely associated with visual field damage in relatively younger male OAG patients. This suggests that anti-oxidant therapy may a promising way to combat diseases associated with systemic oxidative stress, as part of individualized medicine in certain patients.

The current study was somewhat limited by its nature as a cross-sectional case series enrolling only Japanese subjects, all of whom were currently undergoing treatment with anti-glaucoma medication. Additionally, our finding that IOP contributed positively to wrgc was difficult to interpret. The major type of OAG in Japanese subjects, such as the ones in this study, is NTG, and the use of IOP-lowering medication in these subjects could have biased the results. Nevertheless, this study had a large study population, including 480 glaucoma subjects carefully selected by experienced glaucoma specialists, and used a multiple regression analysis, leading us to consider that our results lend sufficient support to our conclusion. Furthermore, our measurement technique was a notable part of this study. Measurement of oxidative stress markers in the blood is generally not a simple or straightforward process. Many pro- and anti-oxidants are relatively unstable, and a long delay between the collection of blood samples and measurement can affect the results. Here, we successfully used dROM and BAP to assess oxidative stress, taking advantage of the speed and simplicity of these biomarkers, which can provide results within 20 minutes with excellent reproducibility. Uric acid, an important antioxidant, is one of many contributors to BAP. Fang *et al*. reviewed several studies in which low uric acid serum levels were observed in patients with neurodegenerative diseases, suggesting that the antioxidant action of uric acid may play a role in preventing neurodegeneration. Nevertheless, there is a need for future research on uric acid, particularly its levels and actions in the serum, in order to clarify its contribution to BAP and to distinguish its role from that of lipophilic antioxidants, such as alpha tocopherol and ubiquinol, which are also important antioxidants that protect against glaucomatous damage in the retina. In the future, we hope to measure the levels of these antioxidants and analyze their association with glaucoma severity.

In conclusion, the current study produced evidence that systemic antioxidant potential is low in male OAG patients, and that antioxidant potential is positively correlated with wrgc in relatively younger male OAG patients. This lends support to the view that low systemic anti-oxidative levels and elevated oxidative stress are involved in the pathogenesis of glaucoma. Biomarkers of systemic oxidative stress should therefore be regarded as valuable sources of supplementary information in glaucoma care. We hope that our results will lay the groundwork for future research aiming to shed light on new antioxidant treatments for glaucoma.

## Methods

### Subjects

This was a prospective study of 240 consecutive Japanese patients with newly or previously diagnosed bilateral open-angle glaucoma, including 168 patients with NTG and 72 patients with POAG. The subjects were enrolled at the glaucoma subspecialty clinic of Tohoku University Hospital between April 2013 and March 2015. This study followed the tenets of the Declaration of Helsinki and was approved by the Ethics Committee of the Tohoku University School of Medicine (study 2014-1-836). Written informed consent was obtained from all participants before entry into the study. The control subjects were recruited from glaucoma-free patients who visited Tohoku University Hospital for surgical treatment of either unilateral cataract or epiretinal membrane (ERM). We selected the healthy contralateral eye of each subject for inclusion. If both eyes had cataracts or ERM, we selected the eye with better VA.

### Recording clinical parameters

All subjects underwent a complete ophthalmic examination, including measurement of best-corrected visual acuity (log MAR: logarithm of the minimal angle of resolution), axial length, slit lamp biomicroscopy, gonioscopy, funduscopy, optical coherence tomography (3D-OCT 2000; TOPCON Corporation, Tokyo, Japan) measurement of cpRNFLT, and evaluation of the optic disc with a 90-diopter lens by a glaucoma specialist. Goldmann applanation tonometry measurement of intraocular pressure (IOP) was performed without interrupting any ongoing anti-glaucoma therapy. MD was measured with the Swedish interactive threshold algorithm-standard strategy of the 24-2 program of the HFA (Carl Zeiss Meditec, Dublin, CA). The eyes with better and worse MD were both included in the statistical analysis. Subjects were excluded if they had other ophthalmic conditions, such as any other type of secondary glaucoma, high myopia exceeding −8D, or hyperopia above +3D. Subjects were also excluded if they had a history of other conditions known to be associated with high levels of oxidative stress, such as cancer or systemic autoimmune disease. The presence or absence of diabetes, hyperlipidemia, and a current smoking habit were recorded before the samples were collected. The blood pressure of all subjects was recorded (all subjects had well controlled blood pressure).

### Blood sampling and analysis

Blood samples were collected at least 3 hours after the last meal. All analyses were performed with a free radical analyzer system (Free Carpe Diem, Wismerll Company Ltd., Tokyo, Japan). All analyses were performed within 1 hour to prevent the influence of falsely high or low metabolites on the results. The analyses were performed according to the manufacturer’s instructions. Briefly, blood samples were collected in a tube and immediately processed by centrifuge. No serum samples contained any anticoagulants. The dROM test measures the activity in the serum of organic hydroperoxides, which are associated with the level of pro-oxidant free radicals. For the dROM test, 20 µl of sample and an acidic buffer were mixed, and a chromogenic substrate was then added. After automated incubation in the analyzer, the absorbance of the sample at a wavelength of 546 nm was recorded. The results were expressed in conventional units, termed U. Carr. The BAP test measures the potential of a sample of serum to reduce Fe3+ to Fe2+, reflecting the power of the systemic response to oxidative stress. For the BAP test, 20 µl of sample was mixed into a colored solution (at 505 nm) containing Fe^3+^. Antioxidants in the sample caused the Fe^3+^ to be reduced to Fe^2+^, leaving oxidized serum barrier molecules and a decolored solution. After incubation for 5 minutes at 37 °C, the decoloration in the sample at 505 nm was quantifiable, and the results were expressed in µmol/L. This method is the same as that used in previously published reports^13, 15^.

### Estimated RGC counts

Wrgc was calculated according to a previously reported formula^19^. All parameters in the calculation, including HFA-measured MD, OCT-measured cpRNFLT, and age, were measured within 3 months of each other.

### Statistical Analysis

The patients were divided into groups based on sex. Furthermore, the patients younger than 65 years were analyzed separately. Spearman’s rank correlation test was used to evaluate single correlations between oxidative stress parameters and other variables. We used a linear mixed-effect model to investigate MD in all eyes of all patients ≤65 years old. This model was fitted with fixed coefficients (i.e., fixed effects) for age and SAF. The analysis used random intercepts and coefficients for all eyes (with each eye nested with a subject). Nonparametric data were analyzed with the Mann-Whitney U test, which was also used to determine the significance of differences between groups. All data are expressed as the mean ± standard deviation. For all tests, P values of less than 0.05 were considered statistically significant. JMP Pro 11 software (SAS Institute Japan, Inc., Tokyo, Japan) was used to analyze the data.
